# Cancer metabolic features allow discrimination of tumor from white blood cells by label-free multimodal optical imaging

**DOI:** 10.3389/fbioe.2023.1057216

**Published:** 2023-02-01

**Authors:** Maria Mangini, Maria Antonietta Ferrara, Gianluigi Zito, Stefano Managò, Alberto Luini, Anna Chiara De Luca, Giuseppe Coppola

**Affiliations:** ^1^ Laboratory of Biophotonics and Advanced Microscopy, Institute of Experimental Endocrinology and Oncology “G. Salvatore”, Second Unit, National Research Council, Naples, Italy; ^2^ Laboratory of Optics and Photonics, Institute of Applied Sciences and Intelligent Systems, Unit of Naples, National Research Council, Naples, Italy

**Keywords:** polarization-sensitive digital holographic imaging, Raman imaging, circulating tumor cells, liquid biopsy, lipid droplets

## Abstract

Circulating tumor cells (CTCs) are tumor cells that have penetrated the circulatory system preserving tumor properties and heterogeneity. Detection and characterization of CTCs has high potential clinical values and many technologies have been developed for CTC identification. These approaches remain challenged by the extraordinary rarity of CTCs and the difficulty of efficiently distinguishing cancer from the much larger number of white blood cells in the bloodstream. Consequently, there is still a need for efficient and rapid methods to capture the broad spectrum of tumor cells circulating in the blood. Herein, we exploit the peculiarities of cancer metabolism for discriminating cancer from WBCs. Using deuterated glucose and Raman microscopy we show that a) the known ability of cancer cells to take up glucose at greatly increased rates compared to non-cancer cells results in the lipid generation and accumulation into lipid droplets and, b) by contrast, leukocytes do not appear to generate visible LDs. The difference in LD abundance is such that it provides a reliable parameter for distinguishing cancer from blood cells. For LD sensitive detections in a cell at rates suitable for screening purposes, we test a polarization-sensitive digital holographic imaging (PSDHI) technique that detects the birefringent properties of the LDs. By using polarization-sensitive digital holographic imaging, cancer cells (prostate cancer, PC3 and hepatocarcinoma cells, HepG2) can be rapidly discriminated from leukocytes with reliability close to 100%. The combined Raman and PSDHI microscopy platform lays the foundations for the future development of a new label-free, simple and universally applicable cancer cells’ isolation method.

## 1 Introduction

The capability to detect circulating tumor cells (CTCs) in the peripheral blood of cancer patients can be exploited for generating minimally invasive diagnostic and prognostic tools (Alix-Panabieres and Pantel, 2016). CTCs are a subset of tumor cells that have acquired the ability to disseminate from the primary tumor and intravasate to the circulatory system. If isolated from blood, they can be cultured and biologically characterized, and can provide valuable information on tumor development and predict patient survival, as demonstrated in patients with metastatic colorectal, breast, prostate, lung and ovarian cancers (Toss et al., 2014; Rupp et al., 2022). CTC examination in peripheral blood is much less invasive than traditional biopsy, and it can be regarded as “liquid biopsy” with the potential to monitor the disease progression and determine the therapeutic choices in different patients, and so to move personalized medicine another step forward (Toss et al., 2014; Rupp et al., 2022). However, technologies to exploit the biological characterization of CTCs have been challenged by the extraordinary rarity of these cells (a few to hundreds per mL of whole blood) among a large number of erythrocytes and leukocytes and by the difficulty of separating them from the large population of white blood cells (WBCs) in the bloodstream. For these reasons, new approaches are continuously proposed with the aim of improving the identification and isolation of CTCs in sufficient numbers and under conditions that are compatible with their molecular and functional characterization. Currently used CTCs identification approaches relying on either the physical properties of cancer cells or expression of biomarkers on the cancer cell surface. Although not with absolute reliability, several physical properties distinguish CTCs from normal blood cells. These include the larger size of most cancer cells and differences in density, migratory properties, deformability or electric charges (Habli et al., 2020). These methods, consisting of filtration-based system, centrifugations or dielectrophoretic, offer a quick and simple way to isolate CTCs, but often with modest selectivity, due to extremely variable CTC sizes or formation of CTC aggregates. Alternatively, CTCs can be separated by immunoaffinity methods based on the presence of biomarkers on the cell surface. To date, the only Food and Drug Administration–approved platform for CTC isolation is an immunomagnetic-based technology, the CellSearch^®^ (Janessen Diagnostic) (Habli et al., 2020). This approach utilizes capture agent-labelled magnetic beads for selection of CTCs using the detection of the epithelial cell adhesion molecule (EpCam) expressed on the CTC surface. Although this method is the most standardized and is now being tested for clinical applications, it suffers from relatively low sensitivity. Only a fraction of patients with metastatic cancer scores positive for any CTCs. In fact, not all tumor types express EpCam and the EpCam expression can be lost during the epithelial-to-mesenchymal transition in metastatic cancers. For these reasons, the CellSearch^®^ is routinely used only for few selected cancer types as breast, lung and prostate carcinomas (Gorges et al., 2012; Hyun et al., 2016; Habli et al., 2020). Therefore, there is still a great clinical need for having an efficient and rapid capture of the entire diverse spectrum of tumor cells that circulate in the blood, followed by their molecular and functional characterization. A desirable scenario is one in which an unbiased low-cost detection and identification of CTCs is performed in a fast and reliable manner, requiring very few manipulations of the sample (i.e., blood) and in a non-destructive way. To address this need, we firstly identified novel morpho-functional parameters expressed differently in tumor cells and leucocytes to be used for efficient identification of these 2 cell types. Secondly, we proposed correlative imaging technologies allowing the detection of these parameters for fast, sensitive and non-destructive detection of viable cancer cells. To identify suitably distinctive parameters, we have relied on the unique metabolic property of cancer cell including the ability to take up glucose at greatly increased rates (5–10 folds) compared to non-cancer cells (the Warburg effect) and to metabolize glucose in a cancer specific manner (Warburg, 1956; Vander Heiden et al., 2009). The Warburg effect is currently at the basis of prognostic and diagnostic cancer imaging techniques that make use of radio-labelled glucose analogues. Typically [^18^F]2-fluoro-2-deoxyglucose (FDG) intracellular concentration is measured *via* positron emission tomography (PET), and routinely exploited in the staging and evaluation of local diseases. Since the local PET signal from tumor cells is higher than surrounding tissues because of Warburg effect, this allows the localization of the anomalous cells within the body, revealing the presence of metastases and allowing the assessment of the tumor response to the adopted treatments (Lewis et al., 2015). This approach, as well as the use radio-labelled or fluorescent glucose analogues, cannot be employed for the identification of viable CTCs as they require long and toxic sample preparations hindering CTC further molecular and functional characterization.

Herein, we studied the glucose uptake and metabolism by cancer cells using the deuterated Glucose (deut-Glc), that is a glucose isotopologue with deuterium labelling on all its carbons, and Raman-based microscopy. The deuterium labelling can act as high-contrast vibrational (chemical) tag producing a Raman band in the so-called Raman silent zone of biological specimen, i.e., where no endogenous molecules contribute any Raman signal. Thus, deut-Glc and its metabolites can be specifically and sensitively detected by Raman microscopy (RM). Importantly, unlike previously reported glucose analogues, deut-Glc is biochemically indistinguishable from the glucose and can be internalized and metabolized by cells without toxic effects (Lee and Cheng, 2017). It can thus be used to study dynamic processes in living cells *in vitro* and *in vivo* even over long incubation times (Gomathy et al., 2010). As expected, we find that the deut-Glc is internalized by cancer cells at higher rates compared to normal cells and white blood cells, and it is metabolized to form efficiently deuterated roundish cytoplasmic organelles, with a diameter of a few microns, clearly detectable by RM. The Raman spectra as well as the morphological analyses showed that these structures are lipid droplets (LDs). These findings are in line with previously reported studies assessing the increase of LD numbers occurring in cells undergoing neoplastic processes (Petan et al., 2018; Cruz et al., 2020; Petan, 2020). In contrast, lipid droplets are nearly undetectable in WBCs. Thus, the separation based of the Raman detection of C-D signals in the lipid droplets of cancer cells compared to WBCs is in principle feasible. However, this identification cannot be performed by RM at the rate needed for screening millions of cells in few minutes, due to the limited sensitivity of the technique. The large difference in LDs abundance between cancer and WBCs must thus be assessed using a microscopy technique enabling acquisitions at video rate speed. It is known that the LDs are formed by highly packed and ordered arrays of cholesterols, triglycerides and phospholipids and exhibiting specific optical density and birefringence (Reginato et al., 1985; Sturley and Hussain, 2012; Bautista et al., 2014; Ioannou et al., 2019; Wang et al., 2020). In order to detect these optical properties, we used a recently developed polarization-sensitive digital holographic imaging (PSDHI) that allows to measure with high sensitivity and velocity the modification of the state of polarization (SoP) of polarized light forward scattered by the sample and correlated with the Raman signals (Coppola and Ferrara, 2020). Furthermore, since it is grounded on a holographic technique, it is quantitative and therefore comparative studies can be carried out. On the other hand, the technique is fast and in principle millions of cells can be analyzed in a few minutes. We show that using this technique, the amount of LDs in cancer cells can be detected and used to discriminate cancer cells from WBCs with reliability close to 100%, at rates suitable for screening purposes.

**FIGURE 1:**
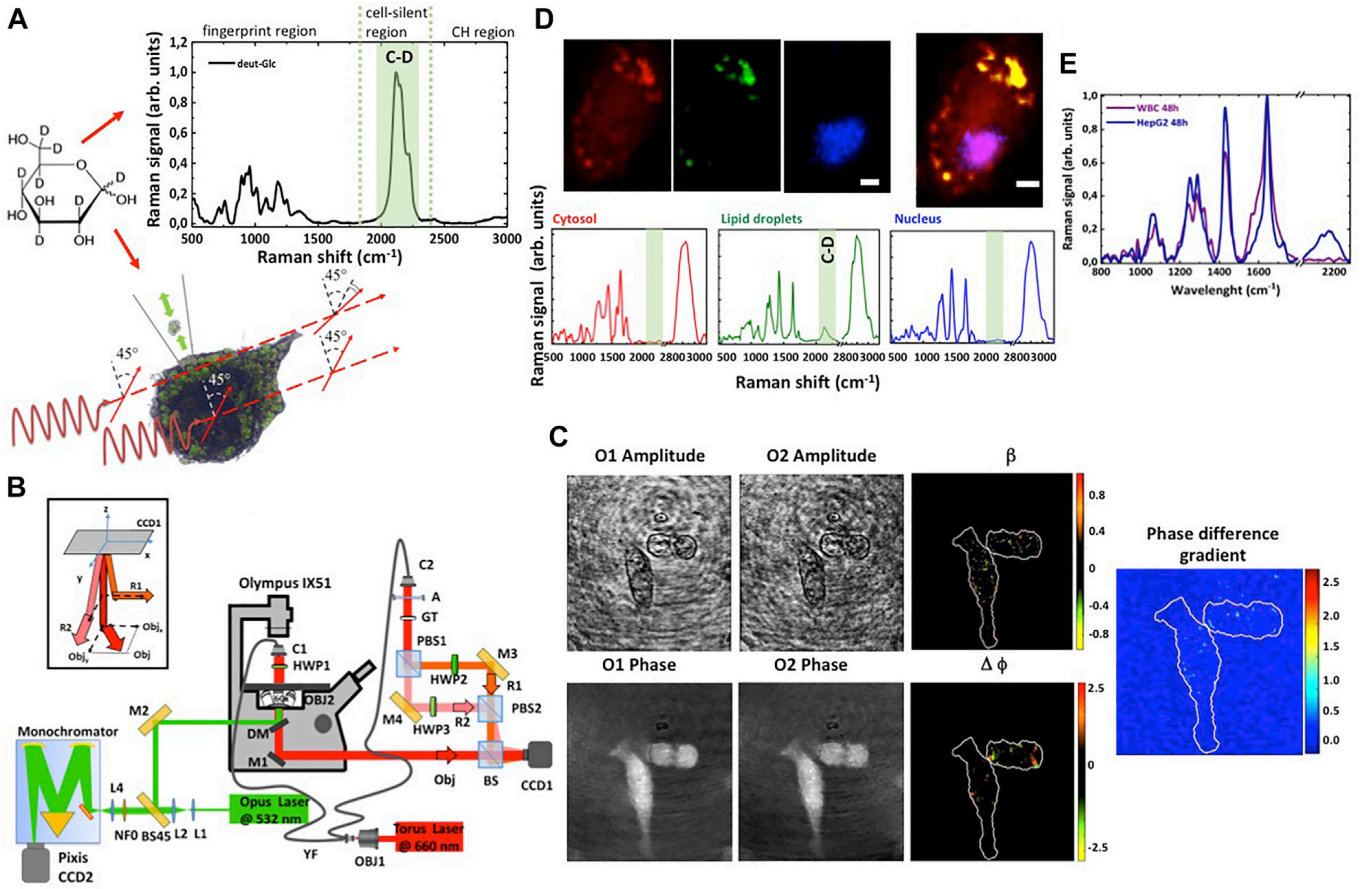
Schematic for the combined imaging by Raman microscopy and polarization sensitive digital holography. **(A)** The Raman spectrum of the isotope labelled glucose (deut-Glc) at 1 M in aqueous solution revealed a Raman band around ∼2,120 cm^−1^. The C-D signal can be used as tracer to visualize the glucose uptake and lipid droplets formation in cancer cells. A change in the polarization of the light is observed only in correspondence of lipid droplets, due to optical rotatory dispersion of these micro-structures. **(B)** Combined experimental setup. **(C)** Reconstructed amplitude and phase maps for the two orthogonal components of the object field and the corresponding amplitude ratio and phase difference maps. The gradient of the phase difference map is reported on the right. The bright spots correspond to strong birefringence variations. Scale bar: 10 μm. **(D)** A representative Raman image of HepG2 cells incubated with deut-Glc. The cytosol is represented in red, the lipid droplets in green and the nucleus in blue. Below, the representative spectra of cytosol, lipid droplets and nucleus. The deuterium signal at 2,120 cm^−1^ is strongly visible in the lipid droplets spectrum. Scale bare: 5 μm. **(E)** Raman spectra of cancer cells (blue line) cells co-cultured with WBCs (purple line). For WBCs, the C-D signal around 2,120 cm^−1^ is completely negligible.

In summary, thanks to combined Raman and PSDHI platform (see Figures 1A, B) we have been able to study the deut-Glc uptake and the consequent formation of LDs at higher rate in cancer cells (both in hepatocarcinoma, HepG2, and prostate cancer cells, PC3), compared to WBCs and even to normal epithelial cells (PNT2). Both cancer cells lines, thanks to internalization and metabolization of deut-Glc, show a strong birefringence signal, which correlates and colocalizes with strong C-D Raman signals, by contrast the WBCs, in the same deut-Glc conditions, do not exhibit measurable levels of LDs. Thus, the detection of these metabolic properties by the combined label-free and non-destructive Raman and PSDHI-based approaches allowed for distinguish tumor cells from WBCs with a high sensitivity and specificity.

## 2 Materials and methods

### 2.1 Sample preparation

Seventy-two hours before the experiment PNT2, PC3 and HepG2 cells were plated in a 24-well plate on CaF2 coverslips at 25,000, 50,000, and 25,000 cells/well, respectively. Details on the cell cultures are reported in Supplementary Materials. Eight hours after the plating, the medium was replaced with a fresh medium made of DMEM containing 5 mM deut-Glc. The day after, 5 mM Glc medium was replaced with a medium containing 2 mM Glc for 1 h. Cells were then washed three times with PBS and cultured for 1 h without glucose or a specific time in a medium containing 25 mM deut-Glc (Li and Cheng, 2014), fresh medium was added every 24 h. For control samples (CTRL), PNT2, PC3 and HepG2 cell cultures were kept for 1 h with no Glc. After the culturing time in deut-Glc, cells were fixed for 10 min at room temperature in 4% (v/v) paraformaldehyde.

White blood cells from blood of healthy donors were obtained by Ficoll-Paque gradient density separation. Cell viability was assessed by trypan blue dye exclusion and found to be >95%. For the WBCs/cell lines co-culture, 24 h before the WBC purification cell lines (PNT2, PC3 and HepG2 at 1,000 cells/well) were plated on CaF_2_ coated with Poly-L-lysine. The day after, WBCs purified from blood were plated together with cell lines already glucose starved (WBCs purified from 10 mL of blood were plated in each well) in a medium containing 25 mM deut-Glc. Fresh medium was added every 24 h and after 48 h cells were fixed for 10 min at room temperature in 4% (v/v) paraformaldehyde. For control experiments, classic Glc was substituted to deut-Glc. Coverslips were then sealed with a CaF_2_ slide, using a common nail polish and Raman and holography characterizations were performed.

### 2.2 Combined Raman and polarization sensitive digital holographic microscope

Raman spectra and image were acquired using an inverted confocal Raman using the laser at 532 nm (50 mW, He-Ne) and a ×60 water immersion objective lens (Nikon, NA = 1.2, WD = 300 μm) (see Figure 1B). The entrance slit of the monochromator was set at an aperture of 100 μm. The optical resolution with such a setup was about 500 nm in x-y plane and about 5 μm along the optical axis. During data acquisition, the laser beam was focused on lipid droplets. However, the scattered light contained average information from different cell sub-compartments (i.e., cytosol, membrane). The laser excitation power on the sample was about 2 mW, Raman spectra were acquired with a 2 s integration time. No cellular photodamage was visible at the employed optical energy. The Raman spectra, after the removing of the cosmic rays, were averaged, and background corrected by creating a baseline with the second Derivative method and then subtracting it with the Peak Analyser function available in OriginLab. The Raman image was acquired by raster scanning the fixed cell through the laser focus with a step size of 0.5 μm, and the acquiring an array of Raman spectra on a selected area (exposure time 1 s per spectrum). A total of 1,500/2,000 spectra per cell were collected. Chemical maps (false colour Raman images) were generated by imaging the DNA peaks at 2,956 cm^−1^ and 790 cm^−1^ (blue signal) for the nucleus, the protein bands at 2,930 and 1,100 cm^−1^ (red signal) for the cytosol and C-D signal at 2,120 cm^−1^ (green signal) for the lipid droplets using HORIBA Scientific Lab Spec six software.

The birefringence analysis of the examined cellular lines was carried out using the laser line at 660 nm (Torus Laser, Max power = 700 mW in CW, coherence length ≈100 m, polarization direction: Vertical, polarization ratio >100:1) shown in Figure 1B. The configuration for recording polarization-sensitive holograms consists of a modified Mach–Zehnder interferometer with two reference waves (R1 and R2) that interfere with the object beam (Obj) (Colomb et al., 2002; Colomb et al., 2003; Palacios et al., 2013; De Angelis et al., 2019; Coppola and Ferrara, 2020). Details are reported in Supplementary Materials. The orthogonality of the two reference beams ensures interference only between the object beam and each reference beam. In addition, controlling the mirrors M2 and M3, the two reference beams can propagate slightly angled with respect the object beam to obtain an off-axis configuration (see the inset in Figure 1B). The two overlapped sets of fringe patterns are acquired by a CCD (CCD1, Thorlabs DCU223M, 1,024 × 768 pixel array; pixel dimensions Δx = Δy = 4.65 μm, fps = 30) and stored for the post elaboration. Finally, to adjust the polarization of the two reference beams, and thus to ensure both the absence of interference between them and a good contrast of the interference fringes with the object beam, two-half wave plate oriented at 0 and 90° are used in the R1 and R2 arms (HWP2 and HWP3), respectively.

### 2.3 SoP evaluation through the numerical reconstruction of the holograms

On the CCD surface, the hologram intensity distribution is:

$$H\left(x,y\right)=\left(Obj+R1+R2\right)∙{\left(Obj+R1+R2\right)}^{*}$$ (1)

where the asterisk indicates the complex conjugate. Assuming that R1 (R2) is linearly polarized along the x (y) direction, and thus taking into account the orthogonality of these beams, i.e. R1⋅R2* = R1*⋅R2 = 0, Eq. 1 can be expressed as:

$$\begin{array}{c} H\left(x,y\right)=\left({\left|{Obj}_{x}\right|}^{2}+{\left|{Obj}_{y}\right|}^{2}+{\left|R1\right|}^{2}+{\left|R2\right|}^{2}\right)\\ +\left({Obj}_{x}{R1}^{*}+{Obj}_{y}{R2}^{*}\right)+\left({Obj}_{x}^{*}R1+{Obj}_{y}^{*}R2\right) \end{array}$$

Thus, the acquired hologram is the superposition of three terms in brackets: the zero-diffraction order, the virtual images, and the real images. These terms are clearly visible performing the Fourier transform of the hologram and reporting the amplitude of this spectrum. In fact, the inclination of the reference beams ensures that the terms relative to the object (second and third term in brackets) are spatially separated from the zero-order term.

The State of Polarization of the Object field can be univocally expressed in terms of the amplitudes (|Obj_x_| and |Obj_y_|) and the phase variations (δx, δy) of the x and y components with the following geometrical parameter structure:

$$\begin{array}{c} \beta =\arctan\left(\frac{\left|{Obj}_{x}\right|}{\left|{Obj}_{y}\right|}\right)\\ ∆\phi =\delta_{x}-\delta_{y}+∆\phi_{R}. \end{array}$$ (2)

The first term is related to the different transmitted intensities of the two orthogonal components and corresponds to the azimuth of the polarization ellipse, whereas the second term contains information on the different optical paths due to the anisotropy of the refractive index of the cell. ${∆\phi }_{R}$ is the phase difference between the two reference beams and can be determinate by a preliminary calibration (Colomb et al., 2003). Thus, to evaluate the SoP of the object beam emersed from the cell, the two orthogonal components along the *x* and *y* directions need to be retrieved. These components can be reconstructed by a spatial filtering algorithm used in the conventional angular-spectrum method (Colomb et al., 2005; Qiu et al., 2015; Han et al., 2017; Dou et al., 2019). In particular, each virtual image of the object beam is properly spatially filtered and multiplied by the relative digitally computed replica of the experimental reference beam, i.e.:

$$\begin{array}{c} \left({Obj}_{x}{R1}^{*}\right)R_{N1}\\ \left({Obj}_{y}{R2}^{*}\right)R_{N2} \end{array}$$ (3)

where RN1 (RN2) is the numerical replica of the reference beam R1 (R2). The method reported in Qiu et al. (2015) was applied to find the replicas of the reference beams as close as possible to the experimental ones. Then, the products of (3) allow shifting the virtual images toward the origin of the Fourier domain and the complex object fields can be retrieved by an inverse Fourier transformation. Hence, a numerical back-propagation can be performed to focus the retrieved complex object field (Ferrara et al., 2015; Qiu et al., 2015; Ferrara et al., 2016). This approach allows to reconstruct the following fields:

$$\begin{array}{c} \left(\left|{Obj}_{x}\right|e^{i\delta_{x}}\right){\left|R1\right|}^{2}\\ \left(\left|{Obj}_{y}\right|e^{i\delta_{y}}\right){\left|R2\right|}^{2} \end{array}$$

Considering that the two reference beams were experimentally adjusted to have the same intensity |R1| = |R2|, the parameters reported in (2) can be easily evaluated.

The single acquired image is a hologram (see Supplementary Figure S1) obtained by the inference of the light emerging from the cells under observation with two orthogonal reference waves. Figure 1C reports an example of retrieved object field in the two orthogonal directions and the corresponding amplitude ratio map, phase difference map and gradient of the phase difference map of the light beam that interacted with the cells under observation.

### 2.4 Principal component analysis of Raman data and the SoP retrieved by holograms

In order to compare results obtained for different cell lines at different glucose incubation time, PCA was performed. This multivariate statistical approach allows extracting the relevant information from the histogram variables and expressing this information as a set of new orthogonal variables useful for identifying similarity/diversity patterns in the observations of the different cells for the different glucose uptake times. In detail, in a Matlab script, from each acquired hologram, both the phase difference map and the amplitude ratio map of the orthogonal components of the light beam emerging from the cells was retrieved. From these maps, only the region containing the cell was selected. Then, histograms variables were generated to summarize the number of pixels at specific grey level values across phase difference and amplitude ratio. By describing these images by their pixel intensity histogram, two arrays of values are obtained and merged to form only one representative vector. Moreover, considering that the phase difference distribution can significantly increase the classification rate (Coppola and Ferrara, 2020), the following additional information were included in this vector: minimum of ∆ϕ, maximum of ∆ϕ, total ∆ϕ and birefringent area normalized to the total area of the cell. The PCA was validated trough the leave-one-out cross validation process and the confusion matrix built up. From this matrix, all the “test characteristics”, such as: sensitivity, specificity, accuracy, positive predictive value (PPV), and negative predictive value (NPV), were evaluated.

The PCA was used for cell Raman spectra classification and validated using the leave-one-out cross validation method. As for holography data, we obtained a confusion matrix that summarizes the correct and incorrect spectra classifications. (Managò et al., 2016).

## 3 Results

### 3.1 Detection of lipid droplets for cancer cell identification

Our aim is to discriminate cancer cells from the WBCs by analysing the cancer cell glucose hyper-uptake and their metabolites, as schematize in Figure 1. To study glucose uptake in cancer cells, we replaced glucose in cell medium to isotope labelled glucose ([D_7_]-glucose). Indeed, the Raman spectrum of the [D_7_]-glucose revealed a Raman band around ∼2,120 cm^−1^, as visible in a representative Raman spectrum reported in Figure 1A, thus occupying the so-called silent spectral region of endogenous molecules (since C-D is physiologically negligible in biomolecules). Thus, deut-Glc and its metabolites can be specifically and sensitively detected and imaged in cells by RM. HepG2 cells were firstly investigated, since they show high rates of Glc uptake and are widely used as model for glucose metabolism studies. HepG2 cells were cultured for 48 h in presence of deut-Glc (25 mM) and then imaged by RM. A representative imaging experiment is shown in Figure 1D. A two-dimensional array of Raman spectra (about 2,500 spectra per cell) was collected on a selected area containing the cell. For each pixel having step size of 0.5 μm, the signal was collected with exposure time of 1 s. Spectra from nucleus, cytosol and granules of the cell exhibited specific bands corresponding to proteins, lipids, carbohydrates and nucleic acids in the fingerprint region. The fingerprint region of the spectrum is characterized by strong bands between 1,100–1,300 cm^−1^ previously associated with Amide III, 1,450 cm^−1^ (C-H deformations in proteins) and 1,660 cm^−1^ (Amide I) (Zoladek et al., 2010). Raman bands associated with amino acids can be visualized at 1,003 cm^−1^ (phenilanalyne), 850 cm^−1^ (tyrosine). The Raman band at 790 cm^−1^ represents mainly DNA present in the cell nucleus. A tentative assignment for these bands was previously reported (Notingher and Hench, 2014; Managò et al., 2018). By imaging the DNA peaks at 2,956 and 790 cm^−1^ (blue signal), the protein bands at 2,930 and 1,100 cm^−1^ (red signal) and C-D signal at 2,120 cm^−1^ (green signal), we reconstructed a chemical map in false colour scale of the cell. In the cell cytosol, subcellular roundish organelles showed a specific signal at 2,000–2,200 cm^−1^ associated with the C-D bands. The presence of the C-D Raman band and the strong band at 2,850 cm^−1^ associated with lipid (cholesterols, triglycerides and phospholipids) together with their typical size of about 1 μm indicates that these round objects are lipid droplets (Li and Cheng, 2014). These observations suggest that in our experimental conditions, as expected, deut-Glc is preferentially used by cancer cells for the lipid synthesis then stored in LDs. As a control, no C-D labelled droplets were detected in the cells cultured with not-deuterated glucose.

We further asked if the hyper uptake and the accumulation of glucose can be used for identification of tumour cells and their discrimination from WBCs. To this end, WBCs were isolated from blood of healthy donors using a Ficoll gradient centrifugation protocol, plated together with HepG2 cells (see Materials and Methods section) and incubated with deut-Glc up to 48 h. Figure 1E reports the Raman spectra acquired from the HepG2 cytosol, in particular from lipid droplets, at Δt = 48 h compared to the signal acquired from WBCs. A very low Raman signal in the spectral region around 2,120 cm^−1^ can be observed in the spectra of WBCs, while, as expected, the C-D band signal is strongly evident in the spectra HepG2 cells. Therefore, the hyper-uptake of glucose in cancer cells and the use of isotope stably labelled glucose could be used as a tracer to visualize by RM the presence of numerous LDs in cancer cells, which can be exploited to distinguish CTCs from normal cells. However, Raman imaging technique generally requires long acquisition times, and it is not always suitable to set up screening strategy.

A standard protocol for detection of LDs in cells it is based on the use of fluorophores (Oil Red O or Bodipy) suitable for selective staining and detection of neutral lipids in cultured cells. However, all the cell membranes generate fluorescent background signals that could mask and, generally, affect the sensitivity and specificity of the LDs’ detection. Herein, we propose to use a specific and selective property of the LDs, that are very dense lipid microstructure with specific optical properties as, for instance, high degree of birefringence (Bautista et al., 2014). Basically, a variation of the cholesterol and phospholipid cell membrane composition induces biochemical and birefringence variation detectable with the polarization sensitive digital holographic imaging. The PSDHI has been demonstrated to be a fast technique allowing the analysis of all the cells in the microscope field of view in a single shot (Coppola and Ferrara, 2020). The PSDHI system has been employed to visualize and monitor the optical state of polarization within a cell as a function of the Glc internalization and to correlate this information with the Raman image. For a fixed optical path length, the observed rotation of the polarization plane of a linearly polarized input beam is proportional to the concentration of the optically active sample. Basically, PSDHI provides vectorial interferograms rendering the spatial distribution of the SoP modified with respect to the homogeneous input because of the interaction with the specimen. Indeed, it provides a non-invasive quantitative estimation of both phase difference and ratio of the amplitudes of the orthogonal components of the light beam through the acquisition of a single holographic image. Figure 1C shows the maps of the phase difference ∆ϕ and the amplitude ratio β allowing to highlight the regions within the cell where an alteration of the SoP is detected. In addition, to stress the phase difference information and to well identify the aforementioned regions on the cell, the norm of the phase-difference gradient (space-variant) ∇∆ϕ was also calculated, as reported in Figure 1C, where:

$$\nabla ∆\phi =\sqrt{{\left(\frac{\partial ∆\phi }{\partial x}\right)}^{2}+{\left(\frac{\partial ∆\phi }{\partial y}\right)}^{2}},$$ (4)

in which $\frac{\partial ∆\phi }{\partial x}$ and $\frac{\partial ∆\phi }{\partial y}$ are the partial derivatives of the phase difference with respect to x and y.

**FIGURE 2:**
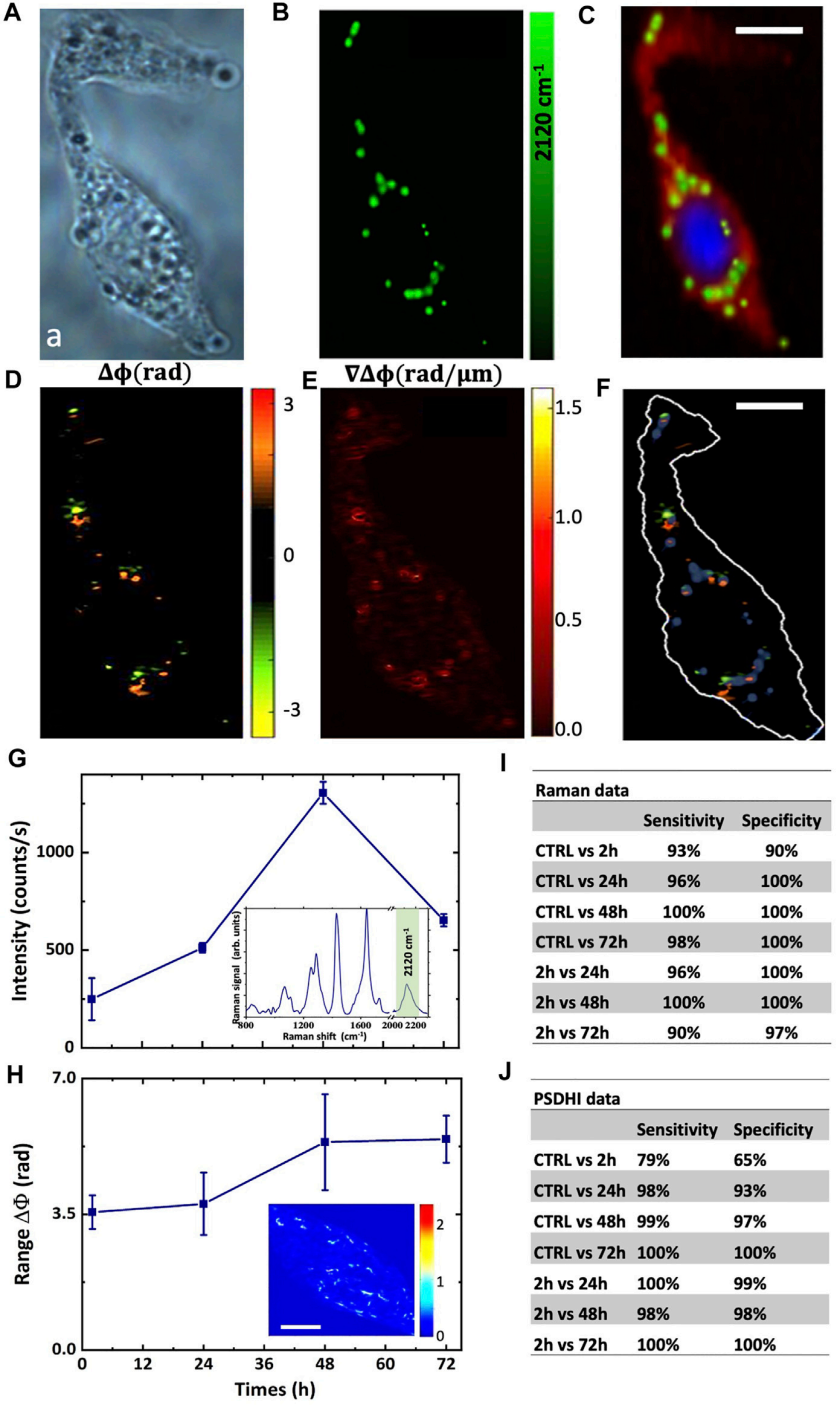
Imaging of the HepG2 cell and D-glucose uptake dynamics. **(A)** Bright field image of the HepG2 cell. **(B)** Raman map of the C-D band signal, and **(C)** reconstructed false colour Raman image using the DNA Raman bands at 2,956 cm^−1^ and 785 cm^−1^ for the nucleus (blue signal), the protein bands at 2,930 cm^−1^ and 1,100 cm^−1^ for the cytosol (red signal) and C-D bands at 2,120 cm^−1^ for the lipid droplets (green signal). The exposure time for each pixel was 1 s. **(D)** Phase difference; and **(E)** the corresponding phase difference gradient maps retrieved by PSDHI. **(F)** Merged image of the Raman map of the C-D band signals and the phase difference map by PSDHI maps, assessing the co-localization of the C-D signal of the lipid droplets and the SoP variation. Scale bar:10 μm **(G)** The graph shows the deuterium peak intensity in HepG2 cells treated with 25 mM deut-Glc for 2, 12, 24, 48 and 72 h. Data are showed as mean ± SD of three independent experiments. Insert shows the mean Raman spectrum of LDs in HepG2 cells treated with 25 mM deut-Glc for 48 h. **(H)** Range of phase difference in radiant for HepG2 cell line treated with 25 mM deut-Glc for 2, 24, 48 and 72 h. Data are showed as mean ± SD of three independent experiments. In the inset, the phase difference gradient is reported. Scale bar is 5 μm. Sensitivity and Specificity obtained from PCA for Raman data set **(I)** and PSDHI data set **(J)**.

The imaging analysis of the space-variant polarization information provided by PSDHI and space-variant biochemical information provided by hyperspectral RM imaging on same cells (HepG2, deut-Glc for 48 h) is reported in Figures 2A–F. By comparing the images acquired by RM and PSDHI, it becomes evident the extraordinary spatial correlation between the C-D Raman signal and the birefringence localization, due to glucose internalization and lipid droplet formation in the analysed cell. Therefore, the biochemical information provided by RM is correlated with polarization variation by PSDHI.

### 3.2 Dynamics of LDs formation in HepG2 cells by Raman and PSDHI

The dynamic of the LDs formation induced by the deut-Glc uptake was assessed, both by Raman spectra and SoP, in HepG2 cells as a function of the deut-Glc incubation period over a time window of 3 days. Cells were cultured for a set of observation periods, Δt, of incubation with deut-Glc, in particular 2, 24, 48, 72 h. As a control (CTRL), HepG2 culture was kept for 1 h in cell medium without glucose. Each set of Raman and PSDHI experiments was repeated three times in order to ensure repeatability and about 90 cells per section were examined. Figure 2G reports the intensity of the C-D band around ∼2,120 cm^−1^ for the Raman spectra acquired from the HepG2 as a function of the incubation times with deut-Glc. The inset shows the average Raman spectrum acquired from the HepG2 lipid droplets at Δt = 48 h. After 24h, we observed the formation of the LDs and consequently C-D signal within the cell became visible. The C-D signal increased from 24 to 48 h of incubation with deut-Glc and then decreased (see Supplementary Figure S2). PSDHI experiments were performed on the same HepG2 cells in the same conditions. A first useful parameter was the spread of phase difference, measured as the maximal range of hologram phase variation ∆ϕ observed on the cell as a function of Δt. The averaged range of ∆ϕ is quantitatively reported in Figure 2H and clearly shows an increase from 2 to 48 h of glucose incubation. After 48 h in glucose, saturation occurs. A visual inspection of the resulting map (see Supplementary Figure S3) shows an increased density of hot-spots characterized by a relevant phase difference ∆ϕ and ∇∆ϕ for incubation times longer than 24 h. These hot-spots appear to be more relevant in the periphery of the cell and in specific accumulation points, which coincides with LDs (Bautista et al., 2014), as shown in Figures 2A–F. Crucially, HepG2 cells were also cultured with non-deuterated Glc (same concentration of 25 mM over an incubation window of 3 days) and, as expected, PSDHI analysis showed same results than those obtained with deut-Glc, confirming that the D-to-H substitution does not affect the uptake dynamics.

In order to define the efficiency and sensitivity of the RM and PSDHI measurements in detecting the LDs formation in cancer cells, a statistical analysis was performed by PCA. As it can be observed from Supplementary Figure S4, The PCA analysis performed on Raman data set provided PC loadings showing spectral differences in the C-D region as well as in the fingerprint region between CTRL cells and cells incubated with deut-Glc at 48 h. The classification sensitivity and specificity were evaluated along the incubation times as reported in the table shown in Figure 2I. Since the Raman spectra provided information not only on C-D bonds but on the overall average molecular state of the cell with sensitivity to its physiological state, it is clear why PCA revealed a variation also among CTRL (starved state) cells and cells fed for 2 h, although not univocally ascribed to the C-D Raman band.

Similar multivariate statistical analysis was carried out directly on both β and ∇ϕ maps. Also, other hologram-inferred parameters were taken into account, as described in Materials and Methods section. The multivariate analysis was performed on the HepG2 data set for each Δt using the first three principal components (pc_1_, pc_2_, pc_3_) accounting for a variance of about 88%, 9%, and 2%, respectively. PCA allowed us to identify patterns of diversity in HepG2 cells treated with Glc for the diverse incubation periods. The corresponding sensitivities and specificities are summarized in Figure 2J, where the results clearly point out an excellent discriminating capability along the various observed Δt with values up to 100%. These data unambiguously demonstrate that PSDHI is able to track the optical perturbation induced on the cells by Glc uptake and metabolism.

Moreover, the RS and PSDHI information correlates with the incubation times and the highest information is detected after 48 h of treatment with deut-Glc.

### 3.3 Figures LDs formation for discrimination of cancer cells and WBCs

**FIGURE 3:**
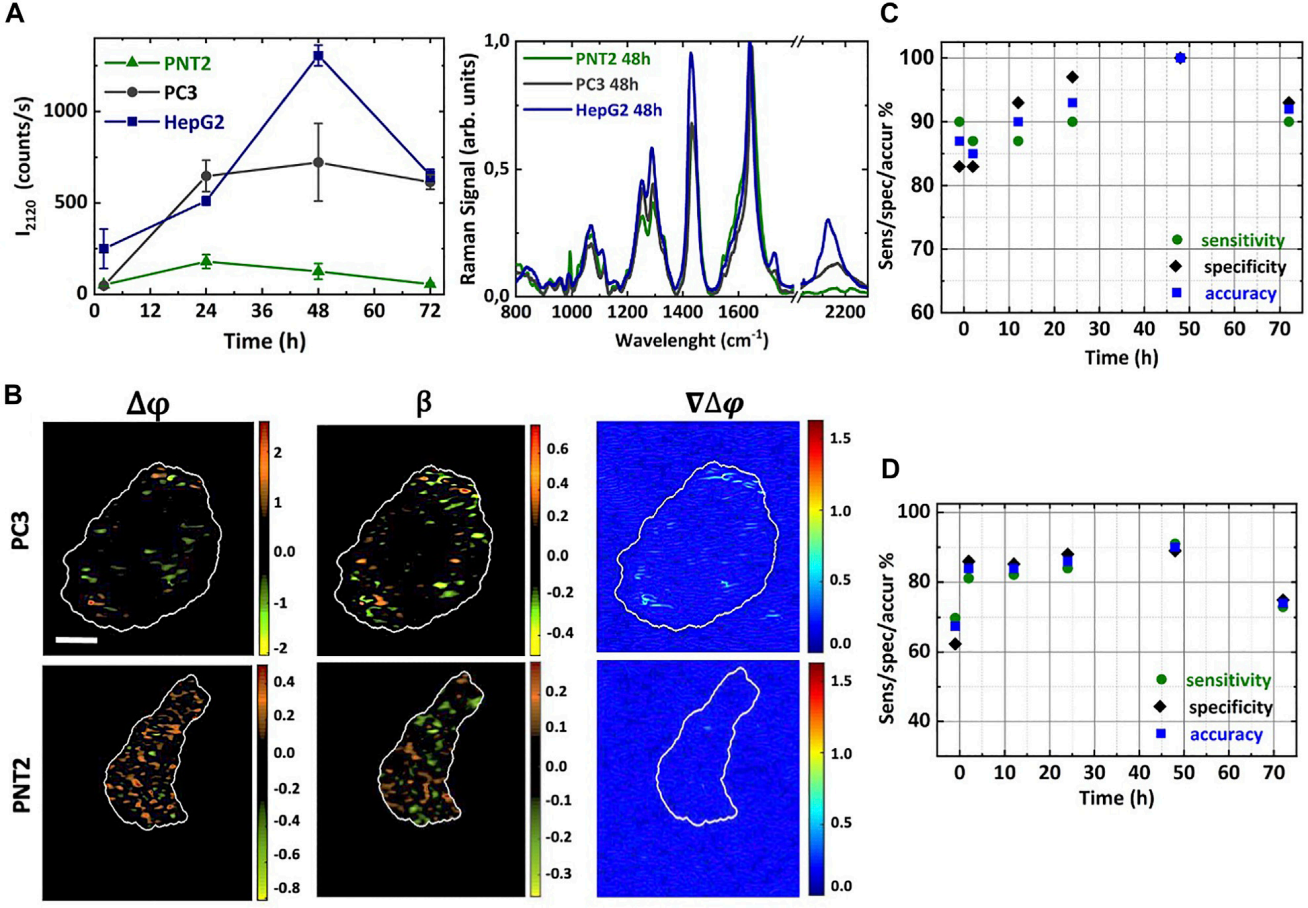
Deut-Glc uptake dynamics in cancer and non-cancer cells. **(A)** Intensity of the C-D Raman bands for normal PNT2 (green triangle) cells, and cancer PC3 (grey circle) and HepG2 (blue square) cells treated with 25 mM deut-Glc for 2, 12, 24, 48 and 72 h. The corresponding mean Raman spectra of PNT2 (green line), PC3 (grey line) and HepG2 (blue line) cells treated with 25 mM deut-Glc for 48 h are additionally shown. **(B)** Phase difference Δϕ, amplitude ratio β and gradient of the phase difference maps ∇Δϕ for cancer PC3 and non-cancer PNT2 cell lines treated with deut-Glc for 48 h. Scale bar: 5 μm. PCA analysis comparing PNT2 and PC3 cell lines. Trend of the sensitivity (green circles), specificity (purple diamonds) and accuracy (blue square) obtained at different glucose incubation times for the Raman data **(C)** and PSDHI data sets **(D)**.

Once the combined approach for Glc-uptake monitoring was demonstrated, RM and PSDHI analysis were carried out on cancer cells and WBCs. However, to define the times and dynamics of differentiation, we preliminary performed an *in vitro* experiment with cancer cells, PC3 (or HepG2) and non-cancer cells, PNT2, deriving from the same prostatic tissue. Deuterated Raman signal was clearly detected only in the cancer PC3 cells, similarly to HepG2 cells (Figure 3A). Indeed, the C-D peak was visible starting from Δt = 24 h of treatment with a maximum at 48 h for cancer cells. The C-D band was clearly lower for the Raman spectra of PNT2 cells, as reported in the spectra of Figure 3A. The same normal and cancer cells were analysed by PSDHI. From a preliminary visual analysis of the obtained results, an increase density of hot-spots with a pronounced phase difference was observed for cancer cells compared to the non-cancer cells (see Figure 3B).

In addition, the number of these hot-spots increased with Δt providing an increased capability of differentiation. As previously described for HepG2 cells, these hot-spots appear to be mainly located in LDs. Rating PC3 and PNT2 results with those obtained with HepG2 cells, the greatest number of such spots was still detected in HepG2 cells, as expected, since they show a more pronounced Glc uptake. An example of β, ∆ϕ, and ∇∆ϕ maps obtained for PNT2 and PC3 cells at Δt = 48 h is illustrated in Figure 3B. Considering the magnified colourbars required for PNT2 imaging, there are evident quantitative differences in the PSDH maps for PC3 and PNT2 cells. In fact, if the same colour bar were used for the PNT2 and PC3 cell, the PC3 maps would appear completely black. Importantly, the ∇∆ϕ maps for the PNT2 cell shows negligible hot-spots, up to 48 h of incubation with deut-Glc, when compared to the background noise level produced by the surrounding bath, pointing out the relevance of Glc hyper-uptake in cancer for a significant optical differentiation. For cancer cells (PC3), we observed an increase in the range of ∆ϕ for increasing Δt. These results confirmed the incidence of Glc hyper-uptake on the birefringence exclusively in the cancer cells. Figure 3C shows the results of the PCA analysis that was carried out on Raman spectral datasets comparing PC3 and PNT2 cells. As a control (CTRL), the cells were kept for 1 h in cell medium without glucose. Confusion matrix was obtained using the leave-one-out cross validation process and the sensitivity, specificity and accuracy were evaluated for all the analysed deut-Glc incubation times. The deut-Glc treatment for 48 h shows the best performances providing 100% accuracy. PCA was also performed on the subsets (HepG2 vs. PNT2) and (HepG2 vs. PC3) as a function of Δt. The results for the cells incubated up to 48 h with deut-Glc and the CRTL cells are reported in Table 1, highlight the optimal discrimination achieved between the three cellular types, based on both the C-D spectral region and fingerprint region even for CRTL cells. The PCA analysis on PSDHI data sets, reported in Figure 3D, showed at Δt = 48 h the highest repeatability and accuracy (about 90%) for the differentiation between normal and cancer cells. In order to rate the Glc-uptake incidence on the holographic analysis, PCA was also performed on the subsets (HepG2 vs. PNT2) and (HepG2 vs. PC3) as a function of Δt. Sensitivities, specificities and accuracies obtained for the most significant cases, i.e. Δt = 48 h and CTRL cells, are summarized in Table 1.

**TABLE 1:** PCA analysis on Raman data set and PSDHI data set comparing the classification sensitivity, specificity and accuracy for HepG2 vs PNT2 and HepG2 vs PC3 at 48 h in deut-Glc and for CRTL cells.

**Raman data**			
**+48 h in deut-Glc**	**Sensitivity**	**Specificity**	**Accuracy**
**HepG2 vs PNT2**	**100%**	**100%**	**100%**
**HepG2 vs PC3**	**99%**	**97%**	**98%**
**CRTL**			
**HepG2 vs PNT2**	**93%**	**91%**	**92%**
**HepG2 vs PC3**	**97%**	**95%**	**96%**

**PSDHI data**			
**+48 h in deut-Glc**	**Sensitivity**	**Specificity**	**Accuracy**
**HepG2 vs PNT2**	**98%**	**97%**	**97%**
**HepG2 vs PC3**	**92%**	**86%**	**90%**
**CRTL**			
**HepG2 vs PNT2**	**71%**	**61%**	**67%**
**HepG2 vs PC3**	**75%**	**65%**	**77%**

Basically, at the maximal differentiation reached at Δt = 48 h, in agreement with the PCA performed on Raman data, the technique is able to successfully discriminate the two cancer cell lines from the normal one (with accuracy up to 97%) as well as between HepG2 cells against PC3 cells (about 90% efficiency), only based on the different optical response they manifest as associated to their phenotypic hyper-uptake of glucose. These results, together with the observations deduced from the Raman analysis, confirm that a label-free detection of glucose uptake and their metabolites can be successfully exploited to distinguish cancer cells from non-cancer cells.

**FIGURE 4:**
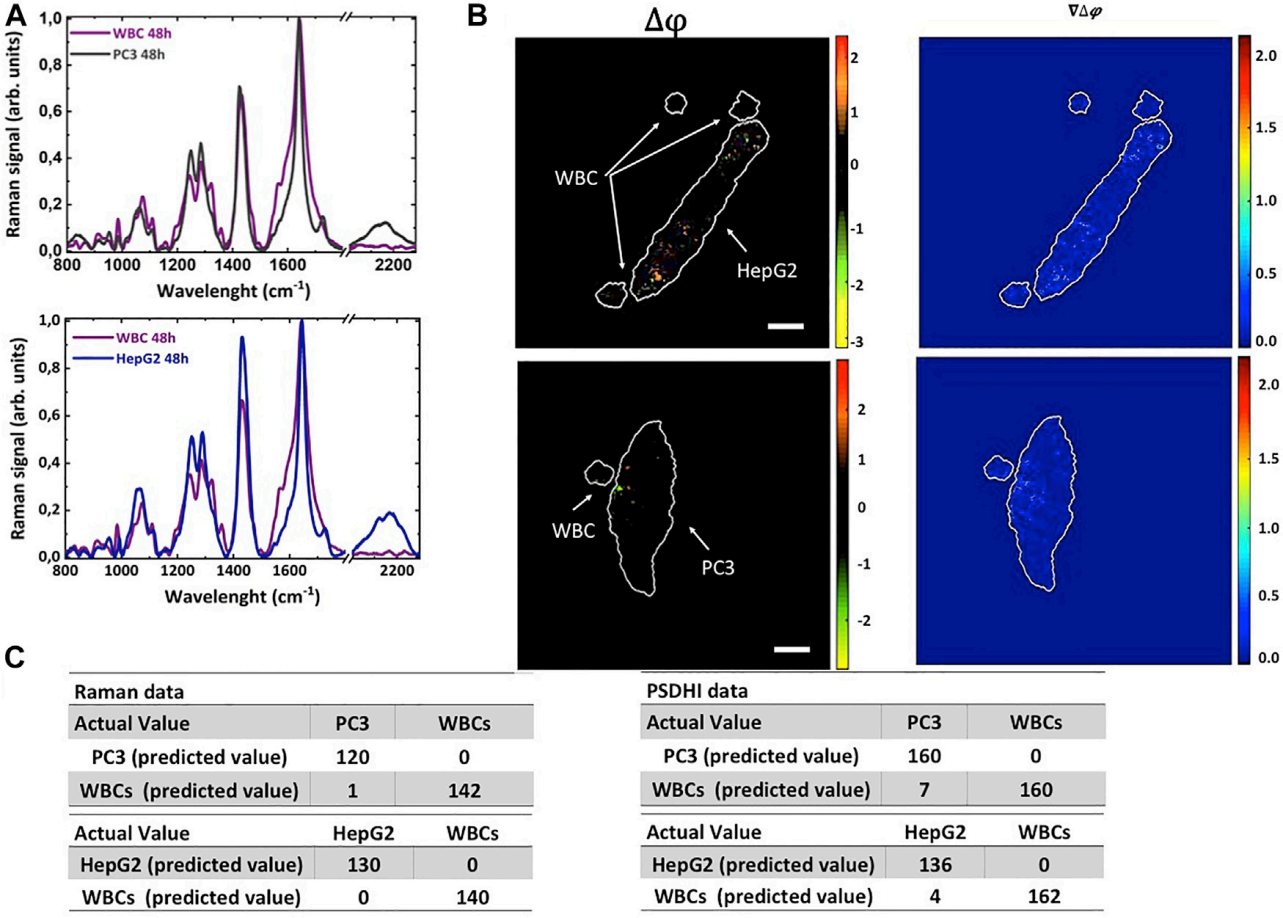
PSDHI and Raman analysis of HepG2 and PC3 cells co-cultured with WBCs isolated from peripheral blood of healthy donors in presence of 25 mM deut-Glc for 48 h. **(A)** Raman spectra of PC3 (grey line), HepG2 (blue line) cells co-cultured with WBCs (purple line in both the graphs). **(B)** PSDHI maps for the co-cultures cancer cells and WBCs. The smaller WBCs do not show significant birefringence variation compared to both HepG2 and PC3 cancer cell lines. **(C)** Results of the classification algorithm by Raman and PSDHI dataset of HepG2 and PC3 cells co-cultured with WBCs.

Finally, WBCs isolated from blood of healthy donors were plated together with PC3 cells or HepG2 cells and incubated with deut-Glc up to 48 h. Figures 4A, B reports the Raman spectra and PSDHI experiments. PSDHI confirms that the WBCs did no show significant variation in the SoP also after 48 h of incubation with deut-Glc, and a very low Raman signal in the spectral region around 2,120 cm^−1^ can be observed. Indeed, the WBCs showed a lower deut-Glc uptake rate and consequently a reduced production of LDs compared to both PC3 cells or HepG2 cells. Furthermore, PSDHI maps for both cancer cell lines revealed several bright spots due to the birefringence of LDs while the co-cultured WBCs appeared completely black.

We performed PCA analysis on the Raman and PSDHI dataset (about 300 cells per experiment were analysed and the experiment was repeated 3 times) to assess the performances of the proposed cancer cells’ identification protocol. The confusion matrix built up on Raman spectral data after 48 h in deut-Glc, based on both fingerprint region and C-D spectral region, led to 100% sensitivity and excellent specificity and accuracy parameters for the discrimination between WBCs and both cancer cells, as shown in Figure 4C. PCA was also performed on datasets obtained by analysing the SoP of WBCs, PC3 and HepG2 cells at the same deut-Glc incubation time (Δt = 48 h) and point out an excellent discrimination capability up to 99% exclusively based on LD birefringence. As reported in Figure 4C, the positive predictive value (PPV) was 100% for both cancer cell types and the negative predictive value (NPV) was 96% and 98%, for PC3 and HepG2 respectively. It is important pointing out that PSDHI results on all cells (around 300) were obtained from images acquired with a fps imposed by the camera used (in our case around 30fps), thus making the proposed approach compatible with screening applications. Further improvements are easily envisaged by enhancing the birefringence signals thanks to the efficient adipogenesis mechanisms in cancer cells, for example by exposing the cells to oleic acids for short periods before screening.

## 4 Discussion

Current methods for cancer cell detection and for exploiting their biological characterization remain challenged by the CTC extraordinary rarity as well as the difficulty of separating CTCs from the background blood cell population with satisfactory sensitivity and reliability. Generally, a combination of approaches is used to remove unnucleated cells, enrich the CTCs population and identify/study them (Sharma et al., 2018). However, there is still a great clinical need for having a fast, general, sensitive and non-destructive discrimination of the living CTCs from the WBCs in the blood.

Based on these considerations, here we present a novel strategy to distinguish cancer cells from WBCs relying on detection of specific metabolic features in cancer cells. We find that cancer cells (both hepatocarcinoma, HepG2 and prostate cancer cells, PC3), fed with glucose, generate and incorporate lipids in LDs and cell membranes more efficiently than non-cancer ones. This is in line with previously reported studies assessing that cancer cells contain abundant lipid droplets functioning as reservoirs of energy and molecular components to support cancer development (Hyun et al., 2016; Melo and Weller, 2016; Geng and Guo, 2017; Jin and Yuan, 2020). LDs have been previously proposed as cancer biomarkers and LD-associate proteins are overexpressed in most if not all cancer cells including colorectal, lung, skin, breast, prostate, hepatic, renal, brain, ovarian and cervical (Cruz et al., 2020; Fader Kaiser et al., 2022). In contrast, we find that WBCs are appeared to be the least efficient LD producer among all the tested cell types. WBCs contain few small, nearly undetectable LDs (1-5 LDs/cells) and even if during pathogen invasion or allergic response some class of WBCs can accumulate more LDs, the amount, size and composition of these remain very different from those of cancer cells (Koizume and Miyagi, 2016; Weller, 2016; Onal et al., 2017; Fader Kaiser et al., 2022). Therefore, the LD abundance in cancer cells represents a general parameter that can be used for efficiently discriminating cancer from blood cells (Cruz et al., 2020).

Thus, using an imaging platform combining Raman imaging and PSDHI we successfully tackled the first experimental challenges mandatory for developing such a label-free cancer cell assessment approach based on LDs detection due to Glc hyper-uptake.

The first component, Raman spectroscopy, due to its chemical specificity, is suitable for metabolic studies and for preliminary characterization of LD formation. We have studied the glucose uptake and its metabolites in cancer cells using a glucose analogue (deut-Glc) with a deuterium tag producing a Raman band at about 2,120 cm^−1^. If compared to the classic PET tracers or fluorescent glucose analogues, the deut-Glc has minimal structural modifications that do not affect the internalization and metabolization by the cells, and it can be used to study dynamic processes in living cells (Lee and Cheng, 2017). Raman hyperspectral imaging of HepG2 cells has been used to localize and image the C-D signal starting from deut-Glc uptake after 48 h from the incubation. The localization of the C-D specific signal in granules of about 1 μm in size together with a strong band at 2,850 cm^−1^ associated with lipids suggest that, as expected, glucose uptake mainly contributed to lipid droplet formations (Li and Cheng, 2014). Previous studies have been reported on glucose metabolic incorporation using isotope-labelled glucose for imaging LDs with Raman techniques (Li and Cheng, 2014). However, they did not study the specific glucose uptake activity in cancer and WBCs. Herein, we observed a very low Raman signal in the spectral region around 2,120 cm^−1^ for WBCs, that indeed showed lower rate of LDs production (Melo and Weller, 2016; Weller, 2016; Fader Kaiser et al., 2022).

The second critical component is PSDHI microscopy. Although RM is able to give specific biochemical information about the deut-Glc content and its metabolites in cancer cells, that can be used to differentiate cancer from WBCs or to characterize the chemical composition of the LDs, it is a technique requiring long acquisition times and, as such, unable to analyze 10^7^ cells. This limitation could be partially overcome with different methods, selecting and imaging single Raman bands, improving the Raman signal collection or using CARS, SERS etc are few examples (Eberhardt et al., 2015) and/or combing the Raman analysis with the microfluidics (Schie et al., 2018). Herein, we correlated the Raman information with the birefringence analysis provided by PSDHI. Our previous studies evidenced successfully the possibility, using Raman and PDSHI-based approach, for label-free visualization of biological events such as heparin-induced sperm cell capacitation, through a birefringence and chemical variation due to the sperm cell lipid structure and composition (Colomb et al., 2005; Ferrara et al., 2015). In this study, by quantitative estimation of both the phase difference and the ratio of amplitudes of the orthogonal polarization components of the light beam interacting with the cancer cell, the PSDHI localizes the strongest birefringence variation in the lipid droplets. Indeed, it has been previously reported that the ordered composition of LDs can alter the interaction with polarized light (Reginato et al., 1985; Sturley and Hussain, 2012; Bautista et al., 2014; Li and Cheng, 2014; Ioannou et al., 2019; Coppola and Ferrara, 2020; Wang et al., 2020) and we demonstrated that it can be sensitively detected by PSDHI. The HepG2 cell map by both Raman and PSDHI imaging provided unequivocally correlations between the deut-Glc uptake, the localization of C-D signal in lipid droplets and the space-variant polarization quantitative information provided by PSDHI.

For both cancer cells (hepatocarcinoma HepG2 and prostate cancer PC3 cells), the SoP variation, as well as C-D signal, increased by increasing the incubation time with deut-Glc up to 48 h. After 48 h of treatment in deut-Glc a strongest variation of SoP, localized in lipid droplets and colocalized with the highest C-D Raman signals, is clearly detected. Longer incubation did not further increase SoP and C-D signal, indicating that lipid storage reached a balanced status at 72 h. On the other hand, PNT2 normal cell and WBCs shows negligible birefringence hot-spots and very low C-D signals up to 3 days of incubation with deut-Glc.

After 48 h of incubation in deut-Glc, the PSDHI approach allowed to fast differentiate between WBCs and cancer cells (i.e. the totality of PC3 and HepG2) with PPV of 100% and NPV values of about 97%, exclusively based on LD birefringence. In our strategy, quantitative PSDHI from single holograms could allow a fast screening of millions of cells at the video rate and therefore, could provide a morphology-based enrichment protocol for the CTC isolation that could be used to remove most of the WBCs. A great advantage of PSDHI is that while cell membranes can be labelled with deuterium (cancer and normal cells) or with fluorescent probe, they do not show birefringence, which eliminates a potentially disturbing background signal from WBC membrane. Importantly, PSDHI detection allows good differentiation between WBCs and cancer cells already at t = 0, without any deuterated glucose incubation. Indeed, the birefringence signal depends only from the presence of LDs and not from the C-D signal. Therefore, the cancer cell discrimination from WBCs could be performed avoiding the long incubation with glucose. In this last case, the performances of the LD birefringence detection protocol could be further amplified and accelerated by exposing the cells to oleic acids for short periods before screening, thanks to the efficient adipogenesis mechanisms in cancer cells. On the other hand, Raman spectroscopy could provide a crossed validation of PSDHI isolated cells as well as provide an in-depth biochemical investigation of the cells sorted out by PSDHI approach. Indeed, the high chemical specific nature of the Raman approach (Managò et al., 2016) could be used to further enhance the separation efficiency of the PSDHI pre-filtered cells.

The proposed cancer cell detection method provides sensitive (99%) and specific (99%) identification of cancer cells with the important advantage, compared to CellSearch®-CTC method, of not depending on the expression of specific markers on the cancer cell surface. Indeed, the sensitivity of the CellSearch^®^ system strongly depends on the type of cancer analyzed with values in the range of 50%–60% (Galletti et al., 2014; Satelli et al., 2015). The methods based on the detection of CTC physical properties show sensitivities comparable to our system but with critical limitations for the further analysis of the cell samples (Galletti et al., 2014; Satelli et al., 2015).

The proposed protocol can be applied to several cancer types (Cruz et al., 2020; Fader Kaiser et al., 2022). Indeed, we demonstrated that the efficiency for the identification of PC3 and HepG2 cells from WBCs is comparable, and, in principle it could be translated to other cancer types. Our combined technique deals with the glucose metabolism, the morphology and the biochemistry of cancer cells (Raman fingerprint), therefore taking into account all these parameters we can differentiate between HepG2 and PC3 (two types of cancer cells showing similar glucose metabolism) with high sensitivity and, in principle, we could differentiate between the cancer cells and other rare cells present in the blood. However, further analysis should be done to asses this issue.

The reliability and repeatability of the results obtained lay the foundations for the development of a new label-free cancer cell detection method able of recognizing a viable cancer cell by exploiting its abnormal metabolism characteristics starting from liquid biopsies.

It is important to note that the label-free nature of our technique and the reduced sample manipulation required may allow the subsequent collection and *in vitro* culture of the cancer cells for the evaluation of genetic and biochemical characteristics or for examining the sensitivity to specific drugs by Raman spectroscopy or other biochemical methods. Despite these important results, challenges remain in the realizing higher sorting throughput. For this, it will be required the enhancement of Raman and PSDHI detection sensitivity, as well as the integration of optics, microfluidics, and signal processing.

## Data Availability

The raw data supporting the conclusions of this article will be made available by the authors, without undue reservation.

## Abbreviations

CTCs, circulating tumor cells; CTRL, control cells; deut-Glc, deuterated Glucose; DMEM, Dulbecco′s Modified Eagle′s Medium; EpCam, epithelial cell adhesion molecule; Fps, frame per second; LDs, lipid droplets; PBS, Phosphate-buffered saline; PCA, principal component analysis (pc_1_: first principal component, pc_2_: second principal component pc_3_: third principal component); PET, positron emission tomography; PSDHI, polarization-sensitive digital holographic imaging; RM, Raman microscopy; SoP, state of polarization; WBCs, white blood cells.
