# Supplementary material for: Cancer metabolic features allow discrimination of tumor from white blood cells by label-free multimodal optical imaging
Source: Front Bioeng Biotechnol. 2023 Feb 1;11:1057216. doi: 10.3389/fbioe.2023.1057216 (PMC9928723; doi:10.3389/fbioe.2023.1057216)
Supplement: Supplementary file 1 [file DataSheet1.docx]

Supplementary Material

# Cell culture and sample preparation

PNT2 (95012613) were from European Collection of Cell Culture (ECACC), PC3 (CRL-1435) and HepG2 (HB-8065) were from American Type Culture Collection (ATCC). PNT2 cells were grown in RPMI supplemented with 10% (v/v) FBS, 2 mM L-glutamine, 100 U/mL penicillin, and 100 μg/mL streptomycin. PC3 cells were maintained in DMEM/F12 supplemented with 10% (v/v) FBS, 2 mM L-glutamine, 100 U/mL penicillin, and 100 μg/mL streptomycin. HepG2 cells were cultured DMEM with 10% (v/v) FBS, 2 mM L-glutamine, 100 U/mL penicillin, and 100 μg/mL streptomycin. All the cells were grown in a humidified atmosphere of 5% CO2 and at 37 °C. White blood cells (WBC) from blood of healthy donors were obtained by Ficoll-Paque gradient density separation. Cell viability was assessed by trypan blue dye exclusion and found to be > 95%.

# Polarization sensitive digital holographic microscope

A linearly polarized optical beam was coupled into a single mode polarization-maintaining optical fiber by an objective lens (OBJ1, Zeiss, 10X, 0.22 N.A.). Then, the laser beam is split through a polarized-sensitive optical fiber coupler (YF, Single Mode Fiber Coupler, 1 × 2, 70/30, FC/APC, Newport) into object and reference beam. The reference beam is collimated (C2, Schäfter+Kirchhoff GmbH 60FC-T-4-M60L-01, Achromat f=60 mm, 0.2 NA, AR 400 – 700 nm) and a Glan Thompson polarizer (GT, Thorlabs GTH10-A) assures a fixed linear polarization of 45-degree, while the optical intensity is adjusted by a continuously variable metallic neutral density filter (A). Then the reference beam is divided into two parts with horizontal and vertical polarization (R1 and R2 beams) by a polarized beam splitter PBS1. These two orthogonal reference beams are then recombined by a second polarized beam splitter PBS2.

The object beam is collimated (C1, Schäfter+Kirchhoff GmbH 60FC-T-4-M40L-01, Achromat f=40 mm, 0.3 NA, AR 400 – 700 nm) and the linear polarization is controlled by a half wave plate (HWP1 oriented at 22.5°). The state of polarization of the Object beam can be altered by passaging through the cell and thus by its birefringence/dichroic proprieties. The object beam, collected by an objective lens (OBJ2, 63X, POL, Leica, NA = 0.75), and the two reference beams are then overlapped by a non-polarized beam splitter BS.

# Supplementary Figures


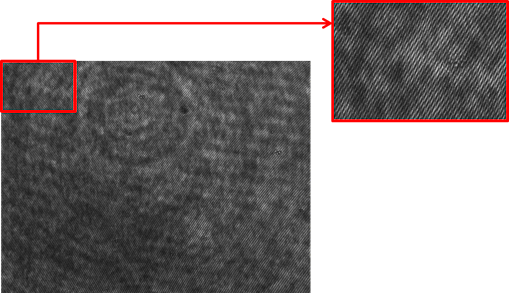


**Supplementary Figure 1.** Polarization hologram. The inset shows the intensity of the fringe pattern.


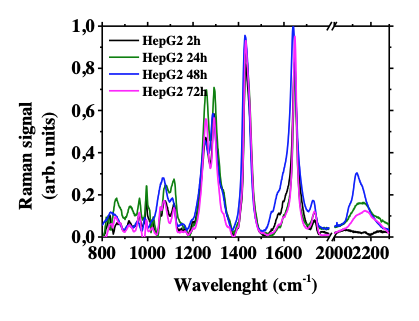


**Supplementary Figure 2.** Raman spectra of HepG2 cells treated with 25 mM deut-Glc fro 2, 12, 24, 48 and 72 h. In each experiment 30 spectra/cells were acquired. Data are showed as mean of three independent experiments.

**
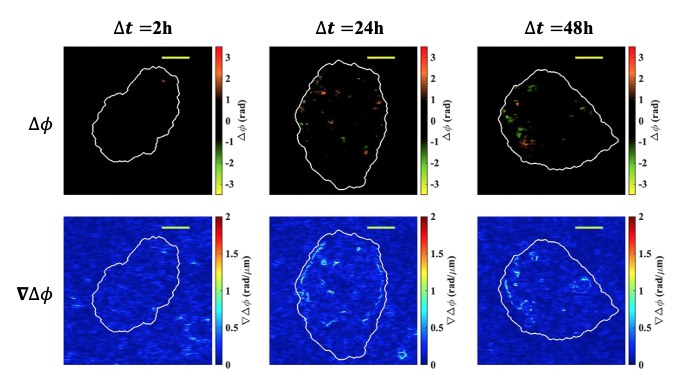
**

**Supplementary Figure 3.** Phase difference map (∆ϕ) and the relative phase-difference gradient ($\nabla\Delta\text{ϕ}$) for HepG2 cell lines for different incubation time with glucose (2h, 24h, and 48h). Scale bar is 5μm.


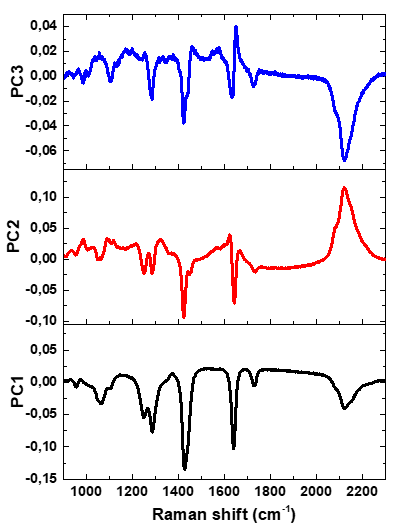


**Supplementary Figure 4.** Loadings obtained for HepG2 ctrl and HepG2 after deut-Glc uptake of 48 hours.
